# Supplementary material for: Beyond Astana: Configuring the World Health Organization Collaborating Centres for primary health care
Source: PLOS Glob Public Health. 2023 Jul 28;3(7):e0002204. doi: 10.1371/journal.pgph.0002204 (PMC10381056; doi:10.1371/journal.pgph.0002204)
Supplement: Attachment — Submitted filename: point by point responses.doc [file pgph.0002204.s001.doc]

**Point by point responses to the reviewers’ comments**

| **Comments** | **Responses** |
| --- | --- |
| Reviewer #1: |  |
| Although the manuscript sums up the revolution of PHC but a more interesting aspect would be to discuss why LMICs are still lacking. It will be amazing if the authors can also summarize the revolution in terms of flow charts so its easier to follow. Good luck with the paper. | We thank reviewer for this feedback. We have summarised the revolution of PHC in figure (Figure 1). Due to limited publicly available information on WHOCCs, this paper has certain limitations why the WHO has not established WHOCCs on PHC in LMICs. We have added this limitation and research implication of this review study. Future primary studies can be conducted to explore the focus of WHOCCs in the development of PHC in LMICs. |
| Reviewer #2: |  |
| The paper is interesting, informative, and highlighting the important issue of WCCs absence from the African region and from LMICs. It would have been interesting if some impact of these centres on the improvement of the health systems or on improving in the number/ support to the country's work force or on capacity development would have been very useful. Authors have mentioned this and absence of information on implementation as their limitation. it is still an important contribution. My few minor comments are as follows; declaration of interest is missing, on page 8 first paragraph please make corrections in Russia and China being mentioned as LMIC, also this does not match with the information given in the Table-1. make same correction in the discussion section as well. On page 16 last paragraph please correct the font size to match rest of the document | We thank reviewer for this feedback. Due to absence of publicly available information, this study has limitation to describe the impacts of WHOCCs on the improvement of the health systems or on improving in the number/ support to the country's work force or on capacity development. We have mentioned this in the limitation in the manuscript and recommended for future primary studies to unpack these issues.  We have corrected minor feedback as suggested. We have included declaration in the submission system (authors have no competing interest to declare). We have corrected the corrected the Russia and China as UMICs as per information in the Table 1 (and also in the discussion section). We have corrected the font size in the manuscript. |
